# Supplementary material for: Patient-Reported Outcome Measures in Patients with and without Non-Expandable Lung Secondary to Malignant Pleural Effusion—A Single-Centre Observational Study
Source: Diagnostics (Basel). 2024 Jun 3;14(11):1176. doi: 10.3390/diagnostics14111176 (PMC11171895; doi:10.3390/diagnostics14111176)
Supplement: Supplementary file 1 [file diagnostics-14-01176-s001.zip › diagnostics-3010040-supplementary.pdf]

Supplementary Material S1
Gold Standard Definitions of NEL
Final NEL status: consensus diagnosis made by two interventional pulmonologists with all data – except the exploratory US-data (USE-SW, B- and M-mode) - available including follow-up four months after first visit.

The diagnosis is either

- Full Expansion (A)
- Probable Expansive Lung (B)
- Definitely NEL (C)
- probable NEL (D)

Entrapped lung scoring on chest x-ray or chest CT scan

- A, Definitely free. Complete apposition of parietal and visceral pleura
- B, Probably free. Apposition of parietal and visceral pleura in most places but some residual pleural fluid
- C, Definitely entrapped. Air separating the visceral and parietal pleura around the lower lobe
- D, Probably entrapped. Some air between visceral and parietal pleura in places around the lower lobe but residual pleural fluid obscuring some areas
- E, Unable to score. Insufficient drainage of pleural fluid to allow designation in one of the prior categories.
